# Parliament and the Professions

**Published:** 1920-07-03

**Authors:** 


### Parliament and the Professions.

### General Medical Council.

The Direct Representation of the General Medical Council Bill was read a second time on June 14, and committed to a Standing Committee.

### Alternative Pensions for Nurses.

Mr. Macpherson informed Mr. Lyle that alternative pensions have not hitherto been allowed for nurses by the Royal Pensions Warrants. The question of extending the benefits of this class of pension to nurses is at present under consideration, but an announcement on the subject could not yet be made.

### Sales of Charity Lands.

In answer to a question as to the present opportunity of the advantageous sales of Charity lands and as to the disposition of the proceeds, the Financial Secretary to the Treasury explained that under Section 24 of the Charitable Trusts Act, 1853, express powers are given to the Charity Commissioners, when authorising the sale of charity land, to give such directions in relation thereto, for securing the due investment of the money arising from such sale for the benefit of the charity, as they may think fit. It is their practice, in the absence of special circumstances justifying a departure from the rule, to require that the proceeds of the sale shall be invested in the names of the Official Trustees of Charitable Funds.

### Public Grants to Voluntary Hospitals.

Mr. Briant asked the Minister of Health if, in the event of grants from public money being made to voluntary hospitals, they will be conditional on the appointment of representatives of the public to the governing bodies. Dr. Addison said that in the event of grants being made such conditions will be attached as are necessary to secure that they are properly expended, but he did not think that the suggestion of representation would be effective for this purpose. In reply to a further question, the Minister said that the question of the extent to which the £250,000 grant of King Edward's Hospital Fund will require to be supplemented from other sources, and the best method of giving such further assistance as is necessary without discouraging voluntary subscriptions, is under consideration, but the whole question involves many complex issues, and he was not in a position to make any further statement.

